# Comprehensive Research of Total Ionizing Dose Effects in GaN-Based MIS-HEMTs Using Extremely Thin Gate Dielectric Layer

**DOI:** 10.3390/nano10112175

**Published:** 2020-10-30

**Authors:** Sung-Jae Chang, Dong-Seok Kim, Tae-Woo Kim, Jung-Hee Lee, Youngho Bae, Hyun-Wook Jung, Soo Cheol Kang, Haecheon Kim, Youn-Sub Noh, Sang-Heung Lee, Seong-Il Kim, Ho-Kyun Ahn, Jong-Won Lim

**Affiliations:** 1 Defense Materials and Components, Convergence Research Department, Electronics and Telecommunications Research Institute, Daejeon 34129, Korea; hujung@etri.re.kr (H.-W.J.); kangsc817@etri.re.kr (S.C.K.); khc@etri.re.kr (H.K.); nohys@etri.re.kr (Y.-S.N.); shl@etri.re.kr (S.-H.L.); sikim@etri.re.kr (S.-I.K.); hkahn@etri.re.kr (H.-K.A.); jwlim@etri.re.kr (J.-W.L.); 2 Korea Multi-purpose Accelerator Complex, Korea Atomic Energy Research Institute, Gyungju 38180, Korea; dongseokkim@kaeri.re.kr; 3 Department of Electrical/Electronic, University of Ulsan, Ulsan 44610, Korea; twkim78@ulsan.ac.kr; 4 School of Electronics and Electrical Engineering, Kyungpook National University, Daegu 41566, Korea; jlee@ee.knu.ac.kr; 5 Department of IT convergence, Uiduk University, Gyeongju 38004, Korea; yhbae@uu.ac.kr

**Keywords:** GaN, SiN, HfO_2_, gate insulator, MIS-HEMT, total ionizing dose effect, γ-ray, radiation

## Abstract

The device performance deterioration mechanism caused by the total ionizing dose effect after the γ-ray irradiation was investigated in GaN-based metal-insulator-semiconductor high electron mobility transistors (MIS-HEMTs) for a 5 nm-thick SiN and HfO_2_ gate dielectric layer. The γ-ray radiation hardness according to the gate dielectric layer was also compared between the two different GaN-based MIS-HEMTs. Although HfO_2_ has exhibited strong tolerance to the total ionizing dose effect in Si-based devices, there is no detail report of the γ-ray radiation effects in GaN-based MIS-HEMTs employing a HfO_2_ gate dielectric layer. The pulsed-mode stress measurement results and carrier mobility behavior revealed that the device properties not only have direct current (DC) characteristics, but radio frequency (RF) performance has also been mostly degraded by the deterioration of the gate dielectric quality and the trapped charges inside the gate insulator. We also figured out that the immunity to the γ-ray radiation was improved when HfO_2_ was employed instead of SiN as a gate dielectric layer due to its stronger endurance to the γ-ray irradiation. Our results highlight that the application of a gate insulator that shows superior immunity to the γ-ray irradiation is a crucial factor for the improvement of the total ionizing dose effect in GaN-based MIS-HEMTs.

## 1. Introduction

Gallium nitride based high electron mobility transistors (HEMTs) have been intensively studied for high frequency, high power, low noise, and aerospace applications thanks to its wide bandgap, high breakdown electric field, high carrier density, and high carrier mobility at the hetero-interface [1,2]. However, there are many dangling bonds at the AlGaN surface, which trap negative charges. The negative trapped charges cause the reduction of the 2-dimensional electron gas (2DEG) density and degradation of the radio frequency (RF) performance that is known as the current collapse effects [3,4]. The lack of the gate insulator results in the high gate current and limitation of the current driving due to the gate Schottky contact in GaN-based HEMTs [5,6,7,8]. To deal with these issues, GaN-based metal-insulator-semiconductor (MIS)-HEMTs have been proposed. The dielectric layer deposited on top of the barrier (or capping) layer improves the radio frequency (RF) performance by diminishing the current collapse when it is utilized as a surface passivation [3,4]. When the dielectric layer is used as a gate insulator, the gate leakage is reduced [5,6] and the current driving capacity is improved [7,8]. However, there are side effects of the gate insulator, which are the transconductance reduction and degradation of RF performance. Therefore, a thin insulator that shows sufficient quality as a gate dielectric layer is required. From this point of view, various gate insulator materials [9,10,11,12,13,14,15] and multi-layered dielectric structures [16,17] have been investigated in GaN-based MIS-HEMTs.

For aerospace applications of the GaN-based MIS-HEMTs, the radiation effects on the device characteristics have been researched for various gate dielectric layers such as SiN [18], SiO_2_ [19], Al_2_O_3_ [20], MgCaO [21], Gd_2_O_3_ [22], and SiN/Al_2_O_3_ multi-layered gate insulators [23]. The radiation hardness of the gate insulator is weaker than that of AlGaN and GaN in GaN-based MIS-HEMTs. The degradation of the dielectric quality and trapped charges inside the dielectric layer (and/or at the dielectric interface), which is the total ionizing dose (TID) effects generated by the radiation, deteriorate direct current (DC) and RF performance of the devices. As the radiation hardness strongly depends on the gate dielectric layer in GaN-based MIS-HEMTs [21,22,23], the device performance degradation mechanism caused by the radiation exposure and the impact of the gate insulator on the device radiation hardness are still not clear in many cases. Therefore, it is necessary to investigate the TID effects induced by γ-ray irradiation on the device characteristics in GaN-based MIS-HEMTs, since this results in the degradation of the dielectric layer quality rather than AlGaN and GaN [24].

In this work, we focused on the TID effects in GaN-based MIS-HEMTs for a 5 nm-thick HfO_2_ gate dielectric layer for the minimization of the transconductance and RF performance degradation. To the best of our knowledge, the γ-ray radiation effect has not been reported in GaN-based MIS-HEMTs for the HfO_2_ gate insulator thus far, even though HfO_2_ exhibited excellent radiation hardness in Si-based electronics [25,26]. After γ-ray radiation, the DC and RF performance deterioration were investigated via electrical characterization. GaN-based MIS-HEMTs for a HfO_2_ gate dielectric layer demonstrated superior immunity to the γ-ray irradiation compared to the SiN gate insulator. In comparison with the SiN gate dielectric layer, when HfO_2_ was employed, the threshold voltage (*V*_TH_) shift (Δ*V*_TH_), transconductance (*g*_m_) maximum deterioration (Δ*g*_m,max_), drain current (*I*_D_), and reduction (Δ*I*_D_) were improved from 0.15 to 0.075 V, 5.42 to 2.27%, and 5.40 to 2.31%, respectively. The pulse-mode stress measurements and extracted carrier mobility (*µ*) behavior revealed that the degradation of the gate dielectric quality and trapped charges inside the gate dielectric layer was the origin of the degradation in device performance in terms of Δ*V*_TH_, Δ*g*_m,max_, and Δ*I*_D_. The RF performance degradation was also improved with the HfO_2_ gate insulator compared to the SiN. The cut-off frequency (*f*_T_) and maximum oscillation frequency (*f*_MAX_) degradation were improved from 36.14 to 13.3% and 34.52 to 10.68%, respectively. Our systematic measurements revealed that the HfO_2_ gate insulator showed excellent immunity to the γ-ray radiation and is suitable for aerospace electronics compared to the SiN dielectric layer. 

## 2. Structure and Fabrication 

**Figure 1:**
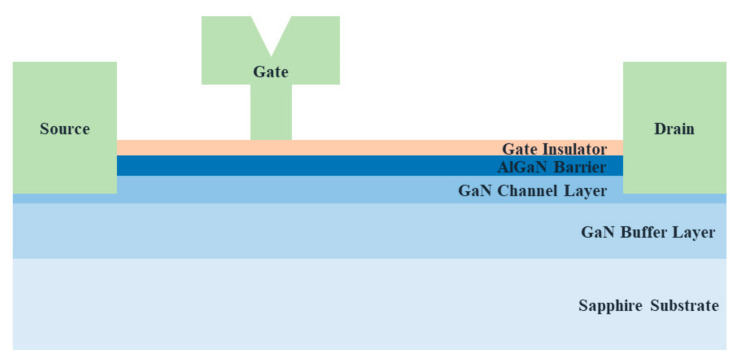
Schematic cross-section of the processed GaN-based metal-insulator-semiconductor high electron mobility transistors (MIS-HEMTs). A 5 nm-thick SiN and HfO_2_ layer were employed for Sample-SiN and-HfO_2_, respectively, as a gate dielectric layer.

The GaN-based MIS-HEMTs were fabricated on a sapphire substrate. The epitaxial layers were grown by metal-organic chemical vapor deposition, which were composed of a 2 µm-thick GaN buffer, 50 nm-thick GaN channel, and 20 nm-thick Al_0.25_Ga_0.75_N barrier layers. Ti/Al/Ni/Au (30/100/30/100 nm) were deposited followed by rapid thermal annealing at 850 °C for 40 s for Ohmic contact formation. For the device isolation, phosphorus was implanted. In order to compare the endurance of the γ-ray radiation and study the device performance degradation mechanism, two different gate dielectric layers (Sample-SiN: SiN 5 nm-thick; Sample-HfO_2_: HfO_2_ 5 nm-thick) were prepared. A SiN layer and HfO_2_ layer were deposited by chemical vapor deposition (CVD) and atomic layer deposition (ALD), respectively. The areas to form the source and drain contact pads were opened through buffered oxide etch. Ni/Au (30/370 nm) were deposited for gate electrode as well as source and drain contact pads. The gate length (*L*_G_), gate width (*W*_G_), and the distance between the source and gate (*L*_SG_), and gate and drain (*L*_GD_) of the processed devices were 0.5, 100, 1, and 3.5 µm, respectively. The radiated γ-ray doses were 0.3, 0.9, 1.8, and 3.6 Mrad(SiO_2_) with the dose rate of 50 rad/s, which were irradiated at the Advanced Radiation Technology Institute. After each γ-ray irradiation, the same device was measured for various electrical measurement. However, there was no hysteresis and/or history effects between the different measurements. Figure 1 shows the schematic cross-section of the fabricated GaN-based MIS-HEMTs. 

## 3. Results and Discussion

**Figure 2:**
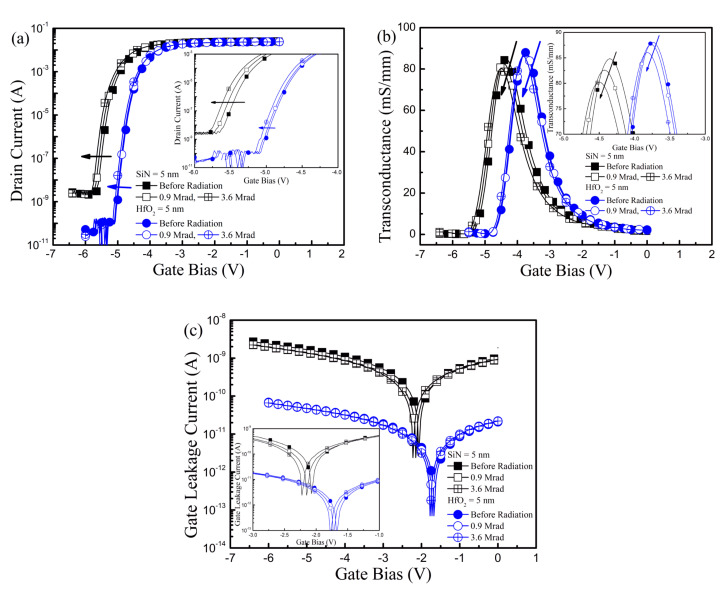
Transfer characteristics of the GaN-based MIS-HEMTs for a 5 nm-thick SiN and HfO_2_ gate dielectric layer before and after γ-ray radiation with the doses of 0.9 and 3.6 Mrad. (**a**) Drain current; (**b**) transconductance; (**c**) gate leakage current as a function of gate bias. Inset in Figure 2a–c shows the zoomed-in drain current at the subthreshold regime, transconductance near the transconductance maximum, and gate leakage current near the gate leakage minimum, respectively.

The alteration of the typical device characteristics produced by the γ-ray irradiation was measured in the GaN-based MIS-HEMTs for the two samples in Figure 2. The drain current and gate leakage current were measured at drain bias (i.e., applied voltage at drain electrode, *V*_D_) = 0.5 V while the gate bias (i.e., applied voltage at gate electrode, *V*_G_) was swept from −6.5 to 0 V and −6 V to 0 V for Sample-SiN and Sample-HfO_2_, respectively. The transconductance was obtained by the derivation of the drain current. The difference of the device properties such as *V*_TH_, *I*_D_, and *g*_m_ in fresh devices (i.e., before the γ-ray radiation) comes primarily from the electrical thickness of the gate dielectric layer [16]. The *V*_TH_ was negatively shifted and *g*_m_ and *I*_D_ were reduced after the γ-ray irradiation in Sample-SiN. Moreover, in Sample-SiN, the Δ*V*_TH_, Δ*g*_m,max_, and Δ*I*_D_ were enlarged with an increase in the irradiated γ-ray doses. In comparison, the device characteristics barely changed after the γ-ray irradiation with the doses of 3.6 Mrad in Sample-HfO_2_, which indicates stronger γ-ray radiation endurance in Sample-HfO_2_. The gate leakage currents were remained almost unchanged after γ-ray irradiation for the two samples. Therefore, the gate electrode was unaffected by the γ-ray radiation.

**Figure 3:**
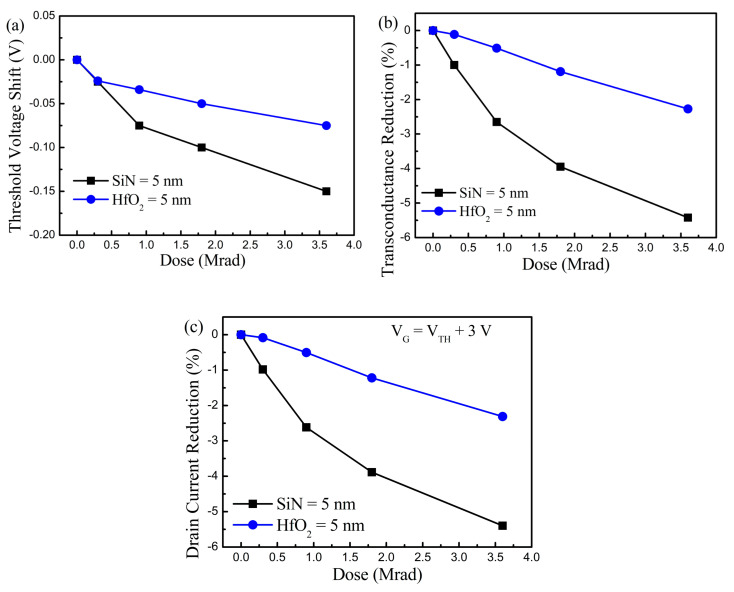
Extracted device parameters before and after the γ-ray irradiation in GaN-based MIS-HEMTs for a 5 nm-thick SiN and HfO_2_ gate dielectric layer. (**a**) Threshold voltage shift; (**b**) transconductance maximum reduction; (**c**) drain current reduction vs. irradiated γ-ray doses.

To quantify the device property change induced by the γ-ray radiation and for the precise comparison of the radiation hardness according to the gate dielectric layer, various device parameters in terms of Δ*V*_TH_, Δ*g*_m,max_, and Δ*I*_D_ were extracted, as shown in Figure 3. The device parameters were achieved at *V*_D_ = 0.5 V for the minimization of the lateral electric field effect caused by the drain bias. The observed overall tendencies of the device parameter variation were identical for the three parameters. However, almost half of the device property alternation was obtained in Sample-HfO_2_ compared to Sample-SiN, despite the epitaxial structure and physical thickness of the gate insulator being the same for the two samples. This phenomenon reflects that the DC performance change resulted from the γ-ray radiation was severely dominated by the gate dielectric property and HfO_2_ showed stronger immunity to the γ-ray radiation than that of SiN. The clues to the device performance variation and the reason for the lower parameter change in Sample-HfO_2_ are described with the pulse-mode measurement results and carrier mobility behavior.

**Figure 4:**
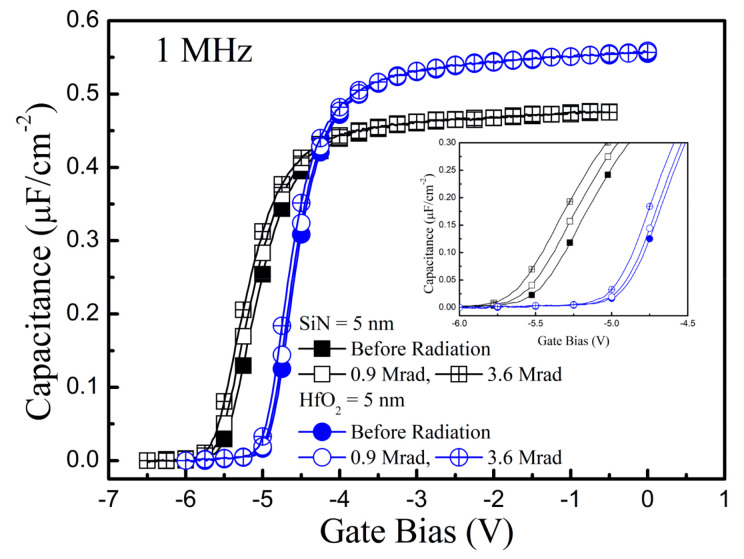
Capacitance-voltage measurements before and after the γ-ray irradiation in GaN-based MIS-HEMTs for a 5 nm-thick SiN and HfO_2_ gate dielectric layer.

In Figure 4, the capacitance-voltage C(V) measurements before and after the γ-ray radiation was conducted at 1 MHz for the two samples. The capacitance was measured while the gate bias was swept like that for the drain current measurement in Figure 2a. After the γ-ray radiation, the capacitance curves were negatively shifted, which was caused by the variation in the channel electron concentration after the γ-ray radiation. However, the shift was less in Sample-HfO_2_ than in Sample-SiN.

**Figure 5:**
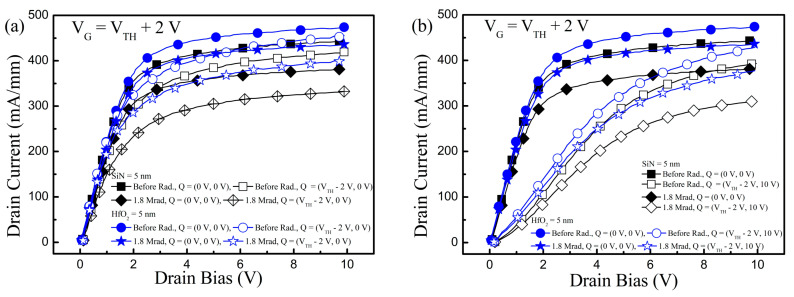

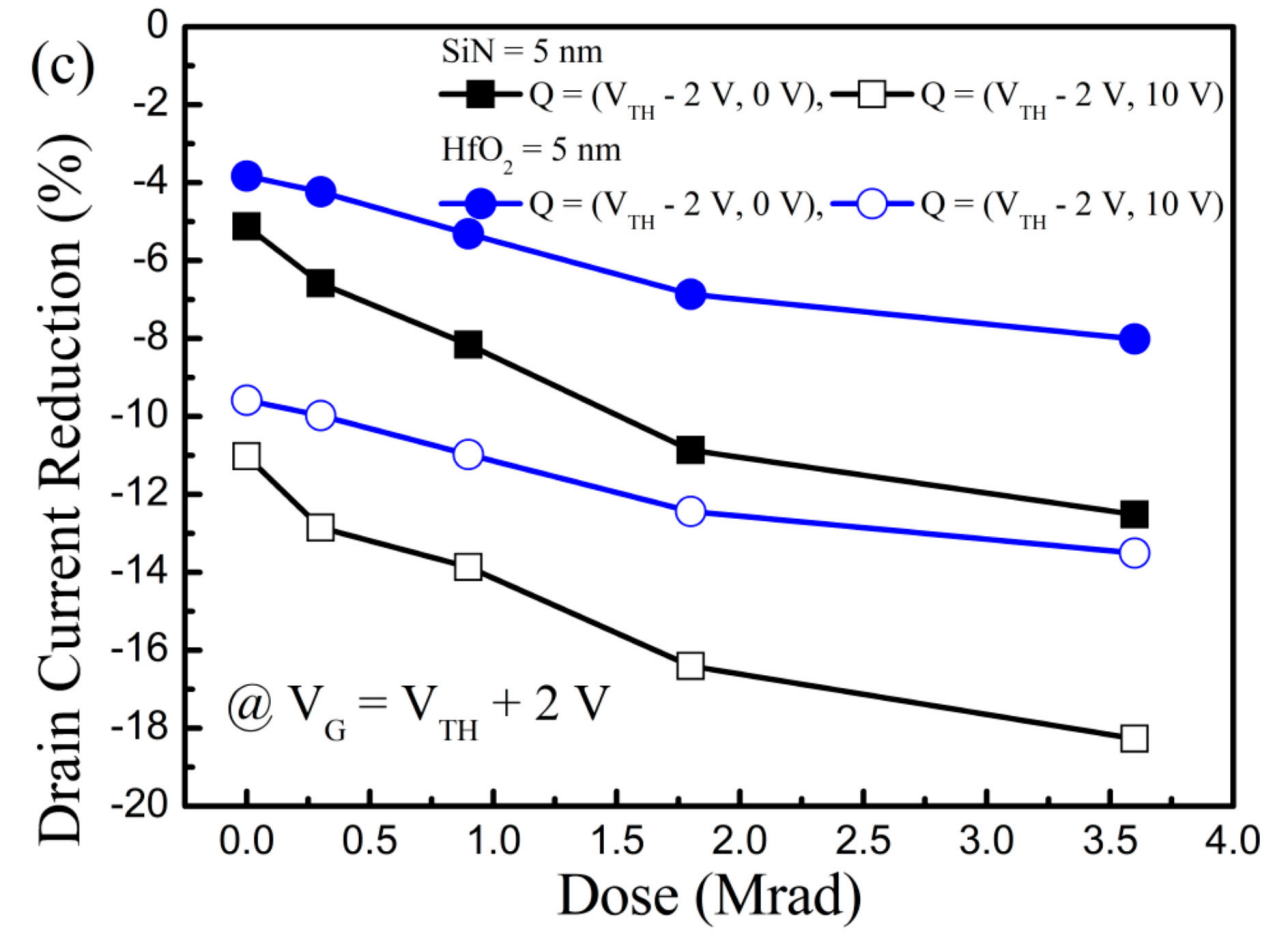
Pulse-mode stress measurement results. Drain current as a function of drain bias with the quiescent bias of: (**a**) (0 V, 0 V) and (*V*_TH_ −2 V, 0 V); (**b**) (0 V, 0 V) and (*V*_TH_ −2 V, 10 V) before and after γ-ray radiation with the irradiated doses of 1.8 Mrad. (**c**) Drain current reduction from the quiescent bias of (0 V, 0 V) caused by the pulse-mode stress with the quiescent bias of (*V*_TH_ −2 V, 0 V) and (*V*_TH_ −2 V, 10 V) vs. irradiated γ-ray doses.

To achieve the key factor of the Δ*V*_TH_ and clues to the stronger radiation hardness of the HfO_2_ layer than SiN, pulse-mode stress measurements [16,27] were carried out, as shown in Figure 5. When the pulse-mode stress is provided, the charges are trapped in the GaN-based MIS-HEMTs [16]. The trapped charges change the carrier density at the AlGaN/GaN hetero-interface and threshold voltage. For the pulse-mode measurements, the drain current was measured at *V*_G_ = *V*_TH_ + 2 V when the pulses applied at drain electrode increased from 0 V to 10 V. For the without stress, gate stress, and gate-and-drain stress condition, (*V*_G_ = 0 V, *V*_D_ = 0 V), (*V*_G_ = *V*_TH_ − 2 V, *V*_D_ = 0 V), and (*V*_G_ = *V*_TH_ − 2 V, *V*_D_ = 10 V) were applied as the quiescent biases, respectively, in the pulse-mode stress measurements. A 2 ms stress pulse was applied, followed by a 0.2 µs pulse to measure the drain current. 

The *I*_D_ was lower than that of the without stress condition when the pulse-mode gate stress or gate-and-drain stress was applied [16,27]. Compared to the gate stress condition, the Δ*I*_D_ was lower under the gate-and-drain stress condition as the impact of traps existed at the GaN channel and/or buffer was included. With an increase in the irradiated γ-ray doses, the Δ*I*_D_ was enlarged. In particular, the Δ*I*_D_ distinction between the gate stress and gate-and-drain stress condition in Sample-HfO_2_ increased to 4.1% after γ-ray irradiation with doses of 3.6 Mrad, and was less than that of Sample-SiN (7.3%). However, the Δ*I*_D_ difference between the gate stress and gate-and-drain stress condition was maintained as the same for both samples. Even the Δ*I*_D_ gap between the gate stress and gate-and-drain stress condition did not increase after the γ-ray irradiation in comparison with the Δ*I*_D_ difference before the γ-ray irradiation. These results verified that the γ-ray irradiation mainly deteriorated the quality of the gate dielectric and generated the charge trapping centers mostly located in the interior of the gate insulator.

The γ-ray irradiation brought out identical results (i.e., negative *V*_TH_ shift) in Sample-SiN and Smaple-HfO_2_, but the occasion was different according to the gate insulator in GaN-based MIS-HEMTs. The silicon-nitride dangling bond defects known as *K* centers [28,29,30] exist in the SiN gate dielectric layer deposited by the CVD system. When the γ-ray is radiated, the neutral *K*^0^ centers trap holes and are converted to positively charged *K*^+^ defects [31]. The charge trapping mechanism of the HfO_2_ dielectric layer deposited by ALD is different from that of SiN. The γ-ray irradiation causes the bond breaking of the HfO_2_. The broken bonds in HfO_2_ result in the oxygen vacancy where there are trap holes [26]. As a result, the 2-dimensional electron gas (2DEG) density is increased by the trapped holes and the *V*_TH_ is negatively shifted. In SiN layer, there are many preexisting point defects (i.e., *K* centers), which can trap holes and be transformed into the *K*^+^ defects [28,29,30,31]. Therefore, the Sample-SiN exhibited larger Δ*V*_TH_ than in Sample-HfO_2_. The degradation of the gate insulator quality and the number of trapped holes were increased when the irradiated γ-ray doses increased, enlarging Δ*V*_TH_ with an increase in γ-ray radiation.

**Figure 6:**
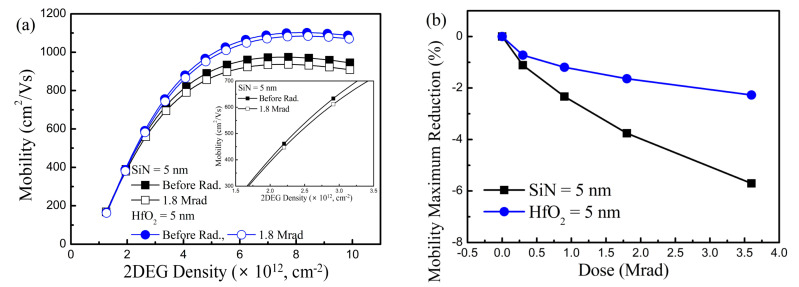
Carrier mobility deterioration according to the γ-ray radiation. (**a**) Carrier mobility as a function of 2DEG density before and after the γ-ray radiation with the doses of 1.8 Mrad. (**b**) Carrier mobility maximum reduction as a function of irradiated γ-ray doses. Inset in Figure 5a shows the zoomed-in carrier mobility behavior at low 2DEG density regime in Sample-SiN before and after the γ-ray radiation with doses of 1.8 Mrad.

To understand the Δ*g*_m,max_ and Δ*I*_D_, the *µ* behavior was extracted before and after the γ-ray irradiation for the two samples, since the *g*_m_ and *I*_D_ are as a function of *µ* (Figure 6). Like the device parameter extraction, a drain bias of 0.5 V was applied to extract the *µ* behavior for the ignorance of the lateral field effect, which changes the carrier concentration and mobility behavior. 

The effect of the parasitic resistance of the access regions (between source and gate and gate and drain) and contact resistance was taken into account to achieve the accurate *µ* behavior under the gated region [32]. To extract the precise mobility behavior under the gated region, we used the following equation:

*μ* = [(*I*_D_/*V*_D_MODIFIED_)*L*_G_]/(*qW*_G_*N*_s_) (1)

where *N*_S_ is the electron concentration estimated by integrating the C(V) curve; *q* is the electron charge; and *V*_D_MODIFIED_ is the drain voltage across the gated channel, as shown in Equation (2):

*V*_D_MODIFIED_ = *V*_D_ − *I*_D_(*R*_ACC_ − 2*R*_C_) (2)

where *R*_ACC_ and *R*_C_ are access region resistance and contact resistance, respectively, which are extracted by the transmission line method (TLM).

The *µ* was deteriorated by the γ-ray radiation and the *µ* maximum reduction (Δ*µ*) was increased with an increase in the irradiated γ-ray doses. The *µ* is reduced from the low 2DEG density regime (inset in Figure 6a), which means that Coulomb scattering between the charges trapped in the interior of the gate insulator and electrons at the AlGaN/GaN interface gives rise to the mobility reduction. The *µ* deterioration ratio was around one-third in Sample-HfO_2_ compared to the Sample-SiN. The tendency of the Δ*µ* corresponded to the Δ*g*_m,max_ and Δ*I*_D_ degradation. Therefore, the *µ* deterioration was the origin of the *g*_m_ and *I*_D_ reduction. 

The sheet resistances of Sample-SiN and Sample-HfO_2_ were extracted by TLM before and after γ-ray radiation (data not shown). The sheet resistance was increased after the γ-ray irradiation due to the Coulomb scattering between the trapped charges inside the gate dielectric layer and electrons at the hetero-interface [23]. When the γ-ray irradiation dose increased, the sheet resistances of the two samples increased. The sheet resistances were 453 (Sample-SiN, before γ-ray radiation), 486 (Sample-SiN, after 3.6 Mrad γ-ray radiation), 451 (Sample-HfO_2_, before γ-ray radiation), and 467 (Sample-HfO_2_, after 3.6 Mrad γ-ray radiation) Ω/□. In Sample-HfO_2_, the deterioration of the sheet resistance was smaller compared to Sample-SiN.

Note that the contact resistance was also extracted via TLM. The extracted contact resistance values were 1.24 (Sample-SiN, before γ-ray radiation), 1.25 (Sample-SiN, after 3.6 Mrad γ-ray radiation), 1.26 (Sample-HfO_2_, before γ-ray radiation), and 1.24 (Sample-HfO_2_, after 3.6 Mrad γ-ray radiation) Ω·mm. Therefore, the obvious damage to the metal electrodes caused by the γ-ray radiation was not achieved. 

Based on the *µ* and sheet resistance deterioration trend with increasing irradiated γ-ray doses and less degradation of the *µ* and sheet resistance in Sample-HfO_2_ than in Sample-SiN, we can inversely verify that (i) the γ-ray irradiation resulted in the degradation of the gate dielectric quality and charge trapping inside the gate dielectric layer, and (ii) less charges are trapped in the HfO_2_ layer than in the SiN layer after the γ-ray irradiation. These results ensure that HfO_2_ shows enough strength to the γ-ray irradiation.

**Figure 7:**
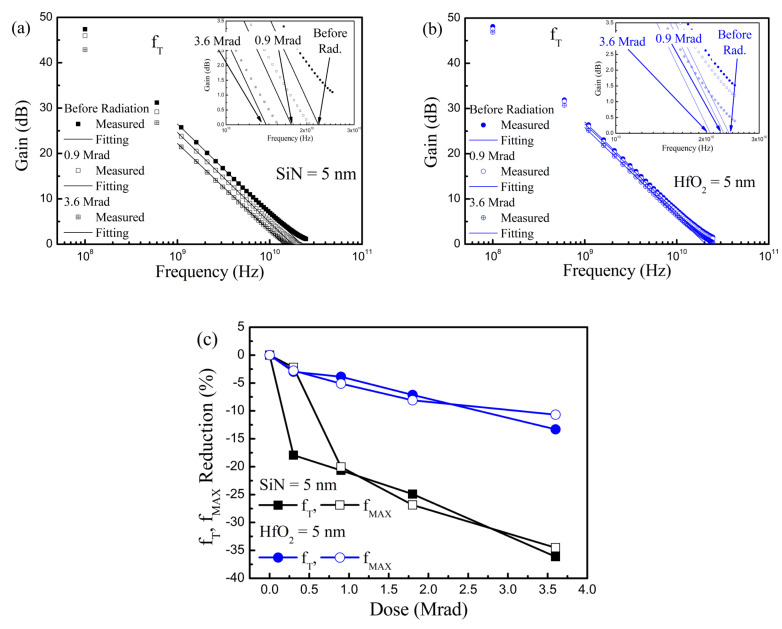
Radio frequency performance degradation induced by γ-ray radiation. Cut-off frequency characteristics before and after the γ-ray radiation in GaN-based MIS-HEMTs for a 5 nm-thick: (**a**) SiN; (**b**) HfO_2_ gate insulator. (**c**) Cut-off frequency and maximum oscillation frequency reduction vs. irradiated γ-ray doses.

In Figure 7, the RF performance degradation generated by the γ-ray irradiation was also researched in GaN-based MIS-HEMTs. For the RF performance characterization, we first measured the *S*-parameter using the network analyzer. For the *f*_T_ extraction, the measured *S*-parameter was converted to the *H*-parameter. Then, we fitted a linear line with a −20 dB slope on the *H*21 curve. The extrapolated point of the linear line to the 0 dB should be *f*_T_. To obtain *f*_MAX_, the measured S-parameter was converted to be maximum stable gain (MSG)/maximum available gain (MAG). We fitted a linear line with a −20 dB slope at stability factor (K) = 1. Like the *f*_T_ extraction, the extrapolated point of the linear line to the 0 dB was defined as the maximum oscillation frequency. 

As we can anticipate from the DC performance alternation, the *f*_T_ and *f*_MAX_ deteriorated after the γ-ray radiation. After γ-ray radiation with doses of 3.6 Mrad, the *f*_T_ and *f*_MAX_ degradation rate was around three times larger when the SiN gate insulator was employed compared to the HfO_2_ gate dielectric layer. Therefore, the RF performance decrease was improved by using HfO_2_ as a gate dielectric layer. In comparison to the DC property deterioration, the RF performance degradation was larger. Note that further investigation is needed to figure out the reason of the relatively large RF performance degradation. One possible scenario is that the RF characteristics of the GaN-based MIS-HEMTs are more sensitive to the frequency response of the radiation-caused traps than the DC properties [24].

## 4. Conclusions

The device performance degradation mechanism of the GaN-based MIS-HEMTs induced by the γ-ray radiation was investigated through electrical device characterization, which was total ionizing dose effect generated at a great part of the gate insulator rather than the AlGaN barrier and GaN layers. According to the γ-ray irradiation, the gate insulator quality was deteriorated and the trapped charges inside the gate dielectric layer were increased. When the γ-ray was radiated, the preexisting defects known as *K* centers were converted to positively charged *K*^+^ defects in the SiN gate insulator. In the HfO_2_ gate dielectric layer, the bond breaking was caused by the γ-ray irradiation and the oxygen vacancies of broken bonds trap holes. As a result, the device performance such as threshold voltage, transconductance, drain current, carrier mobility, and frequency characteristics was deteriorated. However, a huge improvement in the device performance degradation was achieved as the HfO_2_ was employed as a gate insulator due to its superior immunity to the γ-ray radiation. Compared to the SiN gate insulator, the threshold voltage shift, transconductance maximum deterioration, and drain current reduction were improved from 0.15 to 0.075 V, 5.42 to 2.27%, and 5.40 to 2.31%, respectively, when HfO_2_ was used as the gate insulator. The RF performance degradation was also improved by employing the HfO_2_ gate insulator in comparison with the SiN gate dielectric layer. The cut-off frequency and maximum oscillation frequency degradation were improved from 36.14 to 13.3% and 34.52 to 10.68%, respectively.
